# Combining the Fragment Molecular Orbital and GRID Approaches for the Prediction of Ligand–Metalloenzyme Binding Affinity: The Case Study of hCA II Inhibitors

**DOI:** 10.3390/molecules29153600

**Published:** 2024-07-30

**Authors:** Roberto Paciotti, Nazzareno Re, Loriano Storchi

**Affiliations:** Department of Pharmacy, Università “G. D’Annunzio” Di Chieti-Pescara, 66100 Chieti, Italy; nre@unich.it (N.R.); loriano@storchi.org (L.S.)

**Keywords:** machine learning, formula generator, FMO, GRID, scoring function, hydrophobic interactions, metal complexes

## Abstract

Polarization and charge-transfer interactions play an important role in ligand–receptor complexes containing metals, and only quantum mechanics methods can adequately describe their contribution to the binding energy. In this work, we selected a set of benzenesulfonamide ligands of human Carbonic Anhydrase II (hCA II)—an important druggable target containing a Zn^2+^ ion in the active site—as a case study to predict the binding free energy in metalloprotein–ligand complexes and designed specialized computational methods that combine the ab initio fragment molecular orbital (FMO) method and GRID approach. To reproduce the experimental binding free energy in these systems, we adopted a machine-learning approach, here named formula generator (FG), considering different FMO energy terms, the hydrophobic interaction energy (computed by GRID) and logP. The main advantage of the FG approach is that it can find nonlinear relations between the energy terms used to predict the binding free energy, explicitly showing their mathematical relation. This work showed the effectiveness of the FG approach, and therefore, it might represent an important tool for the development of new scoring functions. Indeed, our scoring function showed a high correlation with the experimental binding free energy (R^2^ = 0.76–0.95, RMSE = 0.34–0.18), revealing a nonlinear relation between energy terms and highlighting the relevant role played by hydrophobic contacts. These results, along with the FMO characterization of ligand–receptor interactions, represent important information to support the design of new and potent hCA II inhibitors.

## 1. Introduction

Computational chemistry plays a prominent role in the identification and design of new potential drug-like molecules. Most of the computational approaches used in drug design employ molecular mechanics (MM) methods, which are based on force fields (FFs). These methodologies are generally fast and, within the limit of FF parametrization, disclose an appreciable predictive accuracy. Molecular docking is the most used MM approach in structure-based drug discovery [1], and its accuracy is related to two basic features: (i) the efficiency of the conformational sampling of both ligand and receptor structures, and (ii) the accuracy of the scoring function (SF) adopted to estimate the ligand–receptor (LR) binding energy of the predicted binding poses [2].

Several docking packages, such as DOCK [3], GOLD [4] and LigandFit [5], adopt the so-called force-field-based SFs (FF-based SFs), where the SF is described as a sum of certain non-covalent interaction terms (van der Waals, electrostatic and hydrogen bonding) computed based on a selected FF (the weight factors for all energy terms are equal to 1) [6]. To improve the accuracy of prediction, some additional terms, such as the number of ligand rotatable bonds and ligand logP, can be added to the FF-based SFs leading to the so-called empirical SFs [7]. In these extended empirical SFs, each energy term is weighted through a coefficient obtained via linear fitting of the scoring values to experimental binding data [7]. These functions have been implemented in some currently used docking software, such as Autodock Vina [8] and Glide [9,10].

One of the most important limitations to the employment of molecular docking is represented by the lack of FF parametrization, typically occurring when the structure of either target or ligands includes uncommon functional groups. Moreover, some specific contributions to the ligand–receptor binding, such as induced polarization and charge-transfer (CT) interactions, cannot be modeled using classical MM methods that do not explicitly treat the electronic structure, thus limiting the reliability of the docking method. On the other hand, the quantum mechanics (QM) methods are capable of providing a more accurate energy estimate of non-bonded interactions and polarization and CT effects by explicitly accounting for the electronic structure of the LR adduct. However, the application of QM methods to the study of LR complexes is subjected to a significant computational cost, so these methods are generally used in combination with empirical approaches, for example, to re-score the binding poses predicted through docking calculations (post-docking treatments) [11]. Indeed, many studies were performed to build SFs based on QM methods showing, in many cases, a significant improvement in the correlation with respect to the experimental data [12,13,14,15]. Although the development of multilayered or hybrid QM/MM methodologies has permitted the QM description of the chemically relevant portions of macromolecular systems [12,16,17,18], reducing the computational cost, the lack of parametrization and the possible inconsistencies at the QM/MM boundary still represent common limitations [19].

The fragment molecular orbital (FMO) method is a full QM approach that can be used to investigate the structure and the stability of macromolecular adducts, such as LR complexes, and to predict their binding affinities [20]. In the two-bodies FMO approach (FMO2), the target system is split into several fragments (e.g., one amino acid per fragment), and the total energy is computed as the sum of the fragments’ internal energy and the pair interaction energies (PIEs) [21]. The accuracy of FMO calculations can be increased by adopting the FMO3 and FMO4 approaches [22,23]. It is worth noting that the interfragment interaction energies can be split in several energy contributions, such as the electrostatic (E^es^), exchange repulsion (E^ex^), charge transfer (E^ct^), dispersion (E^disp^) and solvation energy (E^sol^) contributions, by performing the energy decomposition analysis (EDA) that provides for important insights about the chemical nature of the pair interactions [24,25,26].

This decomposing scheme is particularly useful in the study of LR complexes, where one fragment includes the whole ligand, and the sum of its PIEs represents the interaction energy between the ligand and receptor, E^INT^, which can be considered an evaluation of the ligand–receptor binding strength. The EDA of each PIE between the ligand and residues pertaining to the binding site provides relevant details on the nature of LR interactions.

The FMO binding energy, ∆E^FMO^, can be more accurately computed as the difference between the FMO energy of the whole LR complex and the sum of energies of the separated ligand and receptor [20,27]. Indeed, ∆E^FMO^ takes into account the polarization–destabilization and desolvation energies associated with the binding process, providing ideally a more accurate evaluation of the binding strength than E^INT^.

The accuracy of the FMO prediction can be further improved by including hydrophobic interaction contributions, typically playing a relevant role in the stabilization of LR adducts, and not accurately accounting for the QM calculation at the level of theory allowed by the size of the considered system. Recently, we have shown how the FMO description can be coupled with the GRID Force Field calculations [28,29] to define the hydrophobic interaction energies (HIEs) [30,31].

The FMO results were also included in several SFs designed for systems not containing metals where E^INT^ can be considered an evaluation of the enthalpy of the LR interaction, and other terms of the SF are represented by entropic and/or hydrophobic contributions, providing a good correlation with experimental values [32,33,34,35,36].

The relevance of polarization and CT interactions to the stabilization of LR adducts is widely enhanced when metal atoms are present in the active site or part of the ligand/protein structure. Many druggable enzymes contain metal ions in the catalytic site, such as, for instance, Nitric oxide synthase (NOS), Cyclooxygenase (COX), beta-lactamase and Human Carbonic Anhydrase (hCA), to cite some of them. The development of QM-based scoring functions able to correctly estimate these interaction contributions to the binding energy can significantly improve the efficiency of the in silico drug discovery of new metalloenzyme inhibitors.

**Figure 1:**
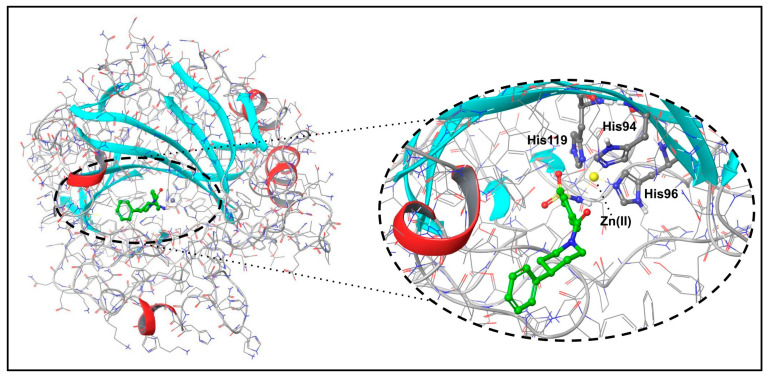
Rendition of the human CA II enzyme co-crystallized with a benzenesulfonamide derivative (PDB ID: 6h2z) (**left**) and structural details of the binding site (**right**).

In this work, a set of congeneric benzenesulfonamide ligands bearing an extended hydrophobic portion and their respective LR complexes with hCA II (Figure 1), retrieved from protein data bank [37,38,39], were selected to develop new SFs based on the combination of FMO and GRID methods.

Several energy terms were combined by adopting two machine learning approaches: the multilinear regression method, widely applied to build SFs, and the formula generator (FG) [40]. To reduce the computational burden and to make our approach applicable to typical in silico drug discovery studies, each LR complex was modeled by assuming the ligand and the CA II residues within a range of 6 Å from all ligands.

The predicted LR binding free energies have been compared to experimental data [37,38,39] by means of a correlation analysis, and the best SF obtained by using the above-mentioned procedure has been compared with other ones developed with similar theoretical approaches, highlighting its strengths and discussing possible improvements.

## 2. Results and Discussion

### 2.1. FMO Binding Energies and Pair Interaction Energies

The FMO method is a powerful tool to investigate the structure and the stability of LR complexes in which the pair interaction energy decomposition analysis (PIEDA) provides for a QM description of the interactions between a ligand and all residues of the receptor, supporting the quantitative structure–activity relationship analysis. The sum of all PIEs between ligand and receptor proteins, E^INT^, has been widely used to estimate LR binding affinities [30,34,41]. Although the FMO scheme is nowadays applied to the study of biomolecular systems, this methodology is characterized by a high computational burden; hence, the chemical nature, the size and the number of the molecular fragments may hamper the application of this methodology. For instance, the use of a high level of theory could be required by the presence of transition metals or heavy elements so that FMO2 calculations of these systems at the RI-MP2 level of theory may be computationally expensive by even using small basis sets. For the same reason, the application of the more accurate FMO3 approach is often unviable. A possible solution to the application of FMO to LR complexes at a moderately reduced computing time is to focus on the FMO description to a reduced model of the system, i.e., the core model, comprising only the ligand and a set of neighbor residues defining the binding pocket. This strategy was already used with the FMO2 approach to estimate the binding energies of human estrogen receptor α with some ligands providing satisfying results [42].

**Figure 2:**
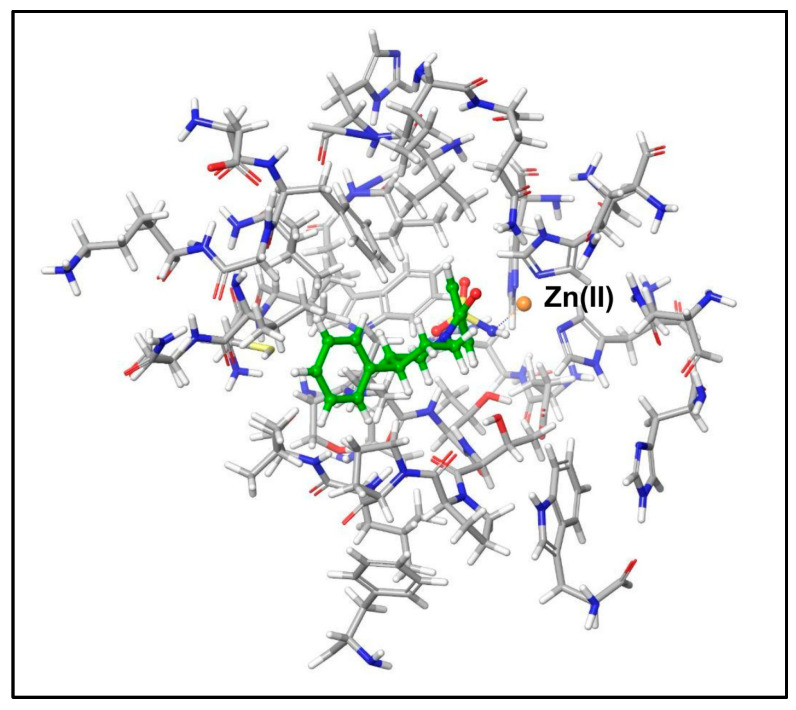
Reduced LR complex of ligand **1**. The N- and C-termini were capped by adding H atoms.

In the present study, we applied the FMO2 method within the core model approach to investigate the binding affinity of nine benzenesulfonamide ligands for the hCA II. By starting from the corresponding X-Ray structures (see Materials and Methods), we included all residues within 6 Å of the ligand to form the core model of each LR complex. For the sake of consistency, the same set of protein residues, i.e., the union of all residues of each reduced binding pocket computed for each LR complex (Table S1), was used to compose the core model of each LR system, consisting of 36 fragments (34 residues, the Zn^2+^ ion and the ligand) (Figure 2).

In the reduced binding pocket, the E^INT^ might be affected by the absence of the polarization effect of the excluded residues and, most importantly, by the capping of single residue termini with H atoms, which changes their chemical nature from amidic CO and NH to aldehyde functions, COH and primary amine, -NH_2_, respectively (Figure 2). Thus, to assess the reliability and the consistency of this approach, we computed the E^INT^ and the single PIEs by performing a preliminary set of calculations at the FMO2 RI-MP2/6-31G//PCM [1] level of theory using the entire LR complexes. As shown in Table S2 and Figure S1a, the E^INT^ computed considering the reduced systems reproduces, in a fair way, the E^INT^ of the entire receptor with R^2^ = 0.90. The correlation is even better when comparing the single PIE between ligands and each residue in both whole and reduced systems as shown in Figure S1b–f (R^2^ ~ 0.999).

Thus, these results clearly indicate that PIEs computed for the reduced LR complexes using an H atom to cap the residue termini reproduced with great accuracy the corresponding values computed for the entire system. This approach can be therefore applied to evaluate the binding strength at the QM level of theory of a great number of binding poses (multi-conformational approach) as the results of molecular docking and molecular dynamics calculations.

The Zn^2+^–ligand interaction provides a relevant contribution to the binding energy (Table S3) and assumes a comparable value for each system between −215.4 and −200.7 kcal/mol. Indeed, all ligands are characterized by the benzenesulfonamide scaffold, which directly binds the Zn^2+^ ion with similar geometrical parameters. Thus, most of the variance affecting the binding geometries and energies for the **1**–**9** LR complexes is expectedly influenced by the remaining part of the ligand scaffold, represented by a mostly hydrophobic aromatic tail. The involvement of this portion in hydrophobic interactions was suggested by PIEDA results, which showed favorable E^disp^ terms in all the examined LR complexes (Table S4).

**Figure 3:**
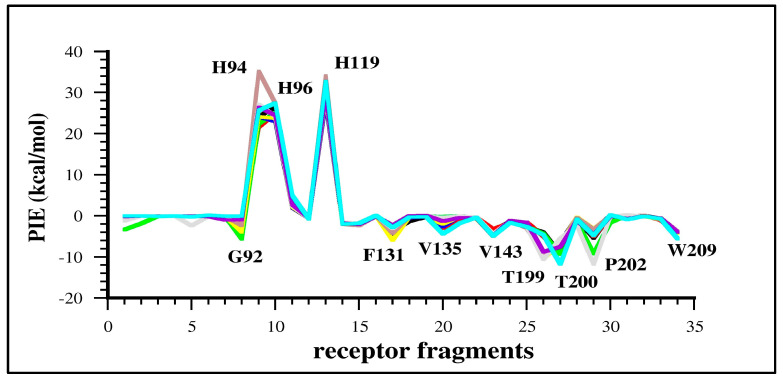
Superposition of PIE graphs computed for all ligands, **1**–**9** (represented by different colors), with the most important receptor residues reported by using the one letter code. The single PIE profiles computed for each ligand are reported in Figure S2a–f (ligands **1**, **2**, **3**, **4**, **5** and **6**, respectively) and S3a-c (ligands **7**, **8** and **9**, respectively).

The PIE charts computed for each ligand provide a clear picture of the key LR interactions. In this case, considering the great structural similarity between ligands, the PIE charts are characterized by very similar shapes, indicating that all binders interact with the same residues with comparable magnitudes (Figures S2a–f and S3a–c). This behavior can be appreciated in Figure 3, where the PIE graphs of all ligands are reported. The PIE analysis per protein residue showed how, with the exception of Gln92 and Thr200, all other negative PIEs involve hydrophobic residues (i.e., Phe131, Val135, Val143, Thr199, Pro202, Trp209) corroborating the important role played by hydrophobic contacts to determine the right placement of the ligand in the binding pocket and, therefore, determining its binding affinity. Indeed, the EDA of these interactions indicates that the most favorable energy term is E^disp^ in agreement with the hydrophobic nature of these contacts.

His94, His96 and His119, which coordinate the Zn^2+^ ion in the catalytic site and place it in close contact to the coordinated benzenesulfonamide moiety, are involved in unfavorable interactions with ligands.

To deepen the different roles played by the benzenesulfonamide function and the hydrophobic aromatic tail, we performed further FMO calculations considering separately F1 and F2 fragments for each ligand. As shown in Figures S4 and S5, the benzenesulfonamide moiety (F1) interacts specifically with His94, His96 and His119 (repulsive contacts) and with Val143, Thr199, Thr200 and Trp209 by means of attractive interactions.

On the contrary, the most relevant interactions established by the F2 fragment are attractive and involve basically hydrophobic residues, such as Trp5, Phe131, Val135 and Pro202, as well as polar His64 and Gln92. This evidence confirms the relevant role played by hydrophobic contacts in LR interactions in this particular system.

The PIEs between ligand and receptor fragments were used to provide several estimates of the receptor–ligand affinity, namely the ΔE^FMO^, F2LE, E^INT^ and FE values (see Materials and Methods) computed at the RI-MP2/6-311G//PCM [1] level of theory for each reduced LR complex (Table 1). Although these energy terms have been shown to reproduce the relative binding affinity of a set of structurally correlated ligands [30,34,41], in this case, the correlation with experimental free binding energies was low with an R^2^ of 0.16, 0.26, 0.12 and 0.25 for ΔE^FMO^, F2LE, E^INT^ and FE values, respectively.

Thus, we hypothesized that the only FMO energy terms are not sufficient to describe the binding free energy of benzenesulfonamide derivatives investigated in this work, and further energy contributions should be considered, such as the hydrophobic interactions.

### 2.2. Scoring Functions

As stated above, the PIEDA clearly indicates that, beside the strong interaction between F1 and Zn^2+^ ions in all examined LR complexes, the interactions of the hydrophobic F2 tail with hydrocarbonic side chains of Phe131, Val135, Pro202 and Leu198 are crucial to determine the binding pose of the benzenesulfonamide derivative and probably provide the highest contribute to the binding energy variance with the considered set of ligands. The strong hydrophobic nature of the F2 interaction with the hCA II residues was suggested by the relevant value assumed by E^disp^ in the corresponding PIEDA.

Thus, we envisioned that the binding energies or affinities calculated via the FMO approach could be complemented by parallel estimates of the LR hydrophobic interactions. The intrinsic hydrophobicity of the ligand and the estimate of the LR hydrophobic interaction were obtained by means of the GRID method (see Materials and Methods).

In order to define the ligand hydrophobicity, we calculated the logP of ligand **1**–**9** (Table 2) and found that ligand 1, the most active compound according to ΔG_exp_, is also characterized by the highest value of logP. Moreover, the HIE values computed with the GRID method also show the most favorable value for ligand **1** supporting the evidence indicated by PIEDA analysis (Table 2).

It is worth noting that, although both logP and HIE can be associated with the hydrophobic features of a ligand, they describe different chemical quantities: logP has been generally used as a measure of lipophilicity (the 1-octanol/water partition coefficient), while HIE is a measure of the interaction energy between hydrophobic regions in the LR complex, and consequently, it is strictly related not only to the ligand lipophilicity but also to its binding pose. Therefore, the combination of FMO terms with logP and HIE values might lead to a function able to correctly reproduce the experimental binding energies of the investigated ligands.

In this view, the general form of the scoring function used in this work is the following one:

ΔG = f(FMO term, logP, HIE)(1)

The considered FMO terms are ΔE^FMO2^, E^INT^, F2LE and FE. LogP, HIEs and the corresponding HIE-Efficiency (HIE-E) values, obtained by dividing the HIE by the number of ligand heavy atoms, are used to describe the hydrophobic contacts. The complete data set used to develop our scoring function, via the described Formula Generator (FG) approach, is reported on Table S4.

The multilinear regression method is widely used to derive SFs and, although they can be characterized by a lack of accuracy, they present the advantage of being easily interpretable just by looking at the physical meaning of either positive or negative weights assigned to favorable and unfavorable energy terms, respectively. We did not find an appreciable correlation between the experimental binding free energies and the calculated energy terms by using the multilinear regression method with our data set, suggesting that eventually these variables might be instead non-linearly dependent.

However, Guareschi et al. recently found a high correlation between experimental binding energy and the linear combination of E^INT^ with logP for several sets of LR complexes [32].

Thus, we deepened the relation between E^INT^ and logP/HIE computed for our data set. We found that the best correlation with experimental values can be obtained by considering only five ligands (**1**, **2**, **3**, **4** and **9**) over nine of the complete data set with an R^2^ of 0.68, computed combining E^INT^ with logP or with HIE, as shown in Figure S6. These results suggest that the linear combination of E^INT^ and logP/HIE can be used to describe the binding affinity only of a limited number of hCA II inhibitors investigated here. In this frame, we opted for a different approach able to identify non-linear dependencies between several quantities and obtain SFs for the prediction of the LR binding affinities within the entire data set.

**Figure 4:**
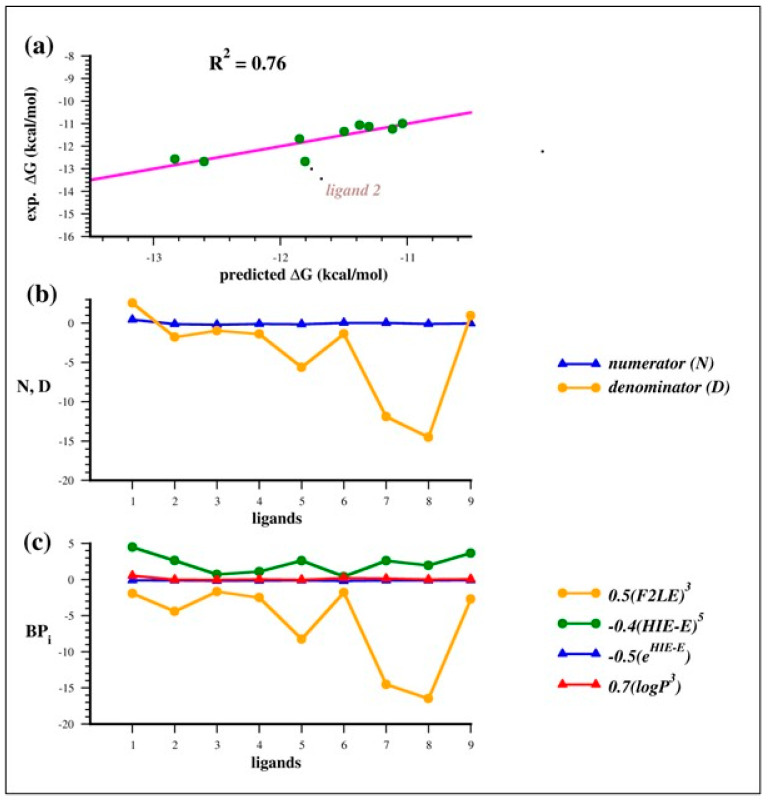
(**a**) Scatter plot of the correlation between the experimental and predicted binding free energy, using Equation (2); (**b**) values of the numerator and denominator of SF (Equation (2)) in blue and orange lines, respectively, computed for each LR complex; (**c**) values of each f(PB) term of SF (Equation (2)) computed for each LR complex.

Indeed, by using the FG approach we found a great number of potential SFs and that one with the highest R^2^, 0.76 and RMSE = 0.34, combines logP, HIE-E and F2LE (Figure 4a):

∆G = −7.4{[0.7(logP)^3^ − 0.5(e^HIE-E^)]/[0.5(F2LE)^3^ − 0.4(HIE-E)^5^]} − 13 (2)

In order to evaluate possible overfitting problems, we adopted the Leave-One Out Cross Validation (LOOCV) approach; the final LOOCV RMSE is 0.31 kcal/mol, which is absolutely comparable to the final model RMSE of 0.34 kcal/mol, indicating that the model is not overfitting.

Looking at the Equation (2), we can easily observe that in the numerator, there are only BPs related to the hydrophobicity and hydrophobic interactions (logP and HIE-E, respectively) and in the denominator, the difference between F2LE, mainly related to polar and electrostatic interactions, and HIE-E, the hydrophobic interaction energy efficiency. This automatically generated formula suggests that the predicted binding free energy is highly related to hydrophobic interactions.

As shown in Figure 4b, the term at the numerator [0.7(logP)^3^ − 0.5(e^HIE-E^)] assumes very small numbers, close to zero, while denominators are larger values. Therefore, the predicted ΔG becomes more favorable (more negative value) if the denominator decreases. Indeed, it is the difference between 0.5(F2LE)^3^ and −0.4(HIE-E)^5^, which are negative and positive values, respectively. These results can be interpreted from a chemical point view as follows: the ligand with a high (more negative) 0.5(F2LE)^3^ term is likely to be a less hydrophobic molecule, since E^INT^ is strictly related to electrostatic and polar interactions, with a consequent lower (less positive) −0.4(HIE-E)^5^ term related to hydrophobic interactions.

Thus, to improve the binding affinity of the benzenesulfonamide derivatives, there should be a certain balance between electrostatic 0.5(F2LE)^3^ and hydrophobic interactions −0.4(HIE-E)^5^ in order to minimize the denominator and maximize the binding affinity (Figure 4c).

This analysis is possible since, unlike many other SFs based on machine learning approaches (e.g., Artificial Neural Network or Random Forest), which are generally not characterized by an easy interpretability, acting mainly as black box [43], the FG approach clearly shows the mathematical relation between the energy terms used to predict the binding free energy, making this machine learning method an interesting option to support the SF development. Moreover, the FG approach, as all other machine learning methods, can be used with any other molecular descriptor and therefore might be applied for the development of new effective SFs to predict the binding free energy.

The prediction accuracy shown by our SF is comparable with that one obtained using a different QM approach used to specifically study the zinc ion-mediated ligand binding, such as hCA and 5-carboxypeptidase inhibitors achieving an R^2^ of 0.8 [44].

**Figure 5:**
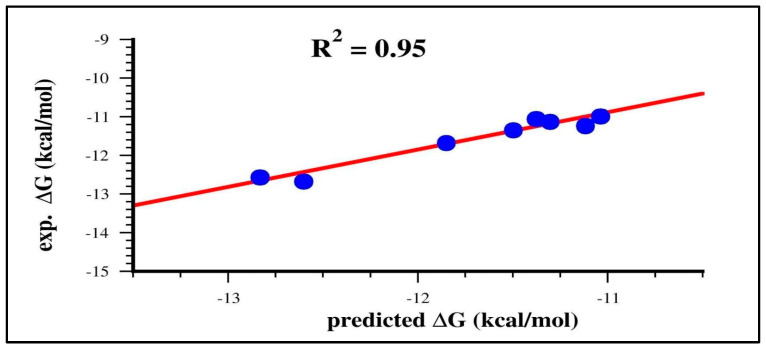
Scatter plot of the experimental and predicted free binding energies (using Equation (2)) excluding ligand **2** from the correlation.

However, it is worth noting that the predicted ΔG value for ligand **2** shows the largest displacement from the correlation line (Figure 4a). Interestingly, removing ligand **2**, the correlation significantly improves with R^2^ and RMSE of 0.95 and 0.18, respectively (Figure 5).

A possible explanation of why ligand **2** shows the highest squared error can be obtained by analyzing its chemical structure. Indeed, the F2 portion of ligand **2** connected to the benzenesulfonamide is characterized by a more polar structure compared to other ligands, which determines, in principle, a better interaction with water molecules. Thus, we hypothesize that the ligand **2** binding pose in the experimental conditions assumed in the measurement of the K_i_ could be influenced by surrounding water molecules and be slightly different from that observed in the crystal structure.

As a final remark, it is important to underline as the FG procedure has two clear advantages with respect to classical machine learning methods: (i) it provides a mathematical formula, a simple relation between the selected features and label; (ii) it can be applied also to a small dataset, being based on simple linear regression. Nevertheless, it should be clarified that, although one can impose some constraints to the FG, the produced mathematical relation is, by construction, obtained minimizing the prediction error, and so its physical meaning should be always assessed. Indeed, the procedure is a computational recipe to automatically build a mathematical formula via human readable construction that relates the features to the label.

Thus, although the limited data set and high similarity between ligands investigated in this work reduce the transferability of our SF to different hCA II binders, we think that this study represents an interesting example of the potentiality of the FG method for the development of an empirical SF. Our future work will be devoted to test the its performance, combined with the FMO/GRID approach, to predict the binding free energy of LR complexes using an extended and more heterogeneous data set.

## 3. Materials and Methods

### 3.1. The FMO Approach

The ab initio FMO approach has been widely applied to study ligand–receptor adducts [34,41] but also to characterize the interactions between biological macromolecules, such as protein–protein [45,46] and DNA–protein [47] complexes and protein domain interactions [48]. Recently, the FMO method has also been used to investigate the reactivity and stability of small metal complexes [49,50].

A ligand–receptor system, where the receptor is a protein, can be split into N fragments where N-1 of them contain one protein residue, while the N-th fragment contains the ligand. The total FMO2 energy, using the polarization continuum method (PCM) [51] to simulate the solvation effect, is computed as the sum of energies of fragments, E’, and fragment pair interaction energies, PIE as follows:

E = ∑E’ + ∑PIE_ij_ (3)

where E’ is the sum of the internal energy (E”) and solvation energy of each fragment. PIE is computed as the difference between the E” values of the *ij* pair and those of the fragments *i* and *j*, including E^sol^, the solvation energy of the *ij* pair interaction and Tr(ΔD_ij_*V_ij_) which is the explicit embedded CT energy:

PIE = (E_ij_” − E_i_” − E_j_”) +Tr(∆D_ij_*V_ij_) + E_ij_^sol.^ (4)

PIE can be decomposed in several terms according to the pair interaction EDA (PIEDA) [23,24] as

PIE_ij_ = E^es^ + E^ex^ + E^disp^ + E^ct^ + E^sol^ (5)

where E^es^, E^ex^, E^ct+mix^, E^disp^ and E^sol^ are the electrostatic, exchange repulsion, charge transfer, dispersion, and solvation contributions, respectively.

The sum of all the PIEs between the ligand (L) and all the protein residues (r), E^INT^, represents an estimation of the ligand–receptor affinity

E^INT^ = ∑PIE_Lr_ (6)

The FMO binding energy, ΔE^FMO^, at variance of E^INT^, includes the destabilization polarization and desolvation energies associated with the binding process [26,27], providing, in principle, a better description of the binding affinity. It can be computed as the difference between the total FMO energy of LR complex and the sum of the total FMO energy of receptor and ligand in the isolated states [26,27], as follows:

ΔE^FMO^ = E_LR_ − (E_R_ + E_L_) (7)

As known, the magnitude of PIE_ij_ is size-dependent, and therefore, a ligand (fragment) with many atoms might have a high E^INT^. This issue can be mitigated dividing the interaction energy by the number of heavy atoms (n), obtaining the fragment efficiency, FE [52]

FE = E^INT^/n (8)

Based on analogy with the ligand efficiency (LE) [53], we introduced, in a previous work, the FMO2 ligand efficiency, F2LE [27], computed as follows:

F2LE = ΔE^FMO2^/n (9)

Notably, as already performed for the classic LE [54], FE and F2LE can be easily combined with other properties, such as lipophilicity, combinations of physicochemical properties, the functional group and entropy contributions to define an efficient SF.

### 3.2. The GRID Approach

**Figure 6:**
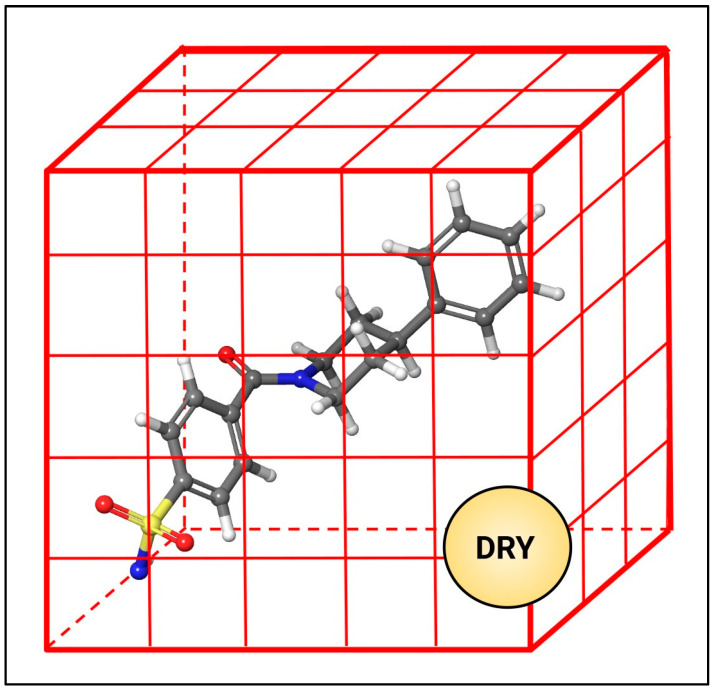
Representation of 3D grid (in red) surrounding a ligand. The hydrophobic interaction energy (HIE) is evaluated by moving the DRY probe along each point of the grid.

In the GRID approach [28], the target structure (e.g., as a ligand or a protein) is surrounded by a three-dimensional grid where a specific probe is moved at each point of the grid (Figure 6). The hydrophobic probe, named DRY, allows for detection of the hydrophobic regions and computing the hydrophobic interaction field (HIF). In detail, it is a neutral probe described as a sort of inverse water molecule, able to establish Lennard–Jones interactions in the same way of the water probe (OH2 probe) but without including the electrostatic interaction term in the energy (Equation (10)) and considering the inverted hydrogen-bond energy to reproduce the energetically unfavorable interaction between the polar parts of the target and the hydrophobic probe. Thus, at each point of the 3D grid, the interaction energy between the DRY probe and an atom of the target is computed as the sum of van der Waals energy (E^VDW^), the inverted hydrogen-bonding energy and entropic (∆S) terms [28]:

HIE = E^VDW^ + EHB + ∆S (10)

∆S is the constant entropic term of –0.848 kcal/mol, added to the total HIE. Indeed, in bulk conditions, the water molecule is assumed to make three hydrogen bonds from the possible four and there are four possible combinations of three hydrogen bonds (1, 2, 3; 1, 2, 4; 1, 3, 4; 2, 3, 4) [28]. The entropy change is computed as

∆S = RTln(4) = −0.848 kcal/mol(11)

where R is the ideal gas constant and T the temperature. Therefore, in GRID, although approximate, the favorable entropic contribution to the binding due to the displacement of one water molecule from a hydrophobic surface (also known as hydration entropy) is taken into account and assumed to be constant.

### 3.3. The Formula Generator (FG) Method—A Machine Learning Approach

Machine learning techniques are currently used in a wide range of scientific areas from chemistry [55,56,57] to material science [40] and far beyond [58]. The range of possible models one can adopt is wide, from the simplest linear regression models to the Deep Learning approaches based on an Artificial Neural Network (ANN) [59]. Different techniques have different advantages and disadvantages; the simplest ones generally guarantee an easy way to interpret the results obtained, although they generally cannot be used with too complex a dataset. For instance, the ANN models can be used in many contexts, and the final results can be a black-box model that is difficult to be practically interpreted. Nevertheless, in the last few years, there has been an enormous step forward in terms of the so-called Interpretable Machine Learning [60]. Thus, nowadays, there are many methods that can be used to obtain insight about the importance of the features in a model and in any case to explore the internal working mechanism also of complex models. However, a mathematical equation connecting two or more quantities has a clear advantage, as it allows for a simple and immediate interpretation of the relation among the quantities involved.

**Figure 7:**
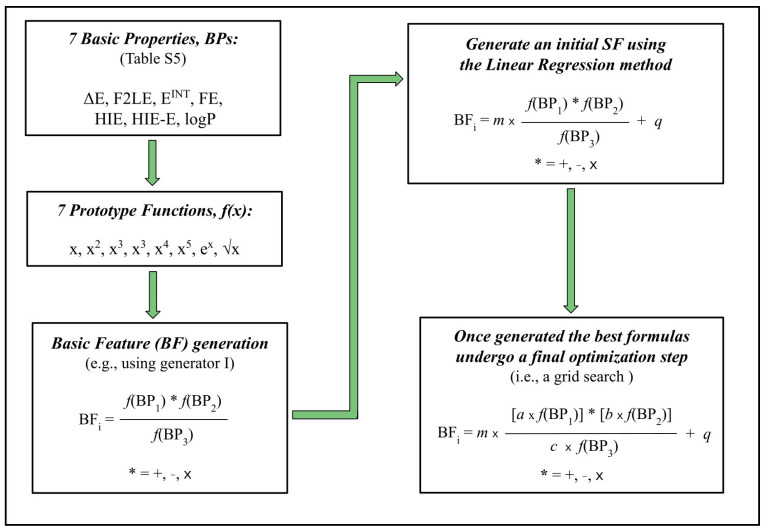
General workflow based on the formula generator approach used to derive an FMO/GRID SF.

The approach we are here proposing (the general workflow is reported in Figure 7) is based on a combination of high-throughput computation and a simple LR model. The basic idea that has been explored by some of us also in a different context [40] is to combine some basic quantities (basic properties, BPs, from now on) to build more complex features that can be used to build a linear regression model.

Once a proper set of BPs has been selected, we choose some prototype functions that are simple analytical operations applied to each BP. In our case, we selected seven prototype functions, f(x), namely x, x^2^, x^3^, x^4^, x^5^, e^x^ and √x, where x is a BP. We impose some constraints, that is rising to even powers, as well as taking the square root, which is applied only to always positively defined BPs. Then, we obtain a final set of basic features (BFs) mixing together the prototype functions, via a combinatorial approach, following two simple rules (i.e., generators):

(i) we sum, subtract or multiply two prototype functions both at the numerator and denominator checking each time that three or four different BPs have been selected. The final general shape of each BF is the following:

BF_i_ = [f(BP_1_) * f(BP_2_)]/[f(BP_3_) * f(BP_4_)] (12)

where * is addition (+), subtraction (–) or multiplication (x).

(ii) We sum, subtract or multiply two prototype functions to build the numerator, and we choose instead a single prototype function at the denominator. So, the general shape of the final set of BFs is the following:

BF_i_ = [f(BP_1_) * f(BP_2_)]/f(BP_3_) (13)

where, once again, * is +, – or x.

From a practical implementation point of view, each BF generator is a Python function producing a set of strings. Therefore, we can easily exploit the Python capability to parse a source code and run a Python expression (code) within a program to compute all the BFs’ values starting from the generated sets of strings. This allows for an easy implementation and plugin of other generators, as well as to easily adopt different sets of basic properties, leaving the workflow unchanged. That is, a new BF generator can be introduced implementing a Python function returning a list of strings, each one being a valid BF.

Clearly, depending on the set of BPs chosen, we will obtain a different set of BFs. In order to choose the optimal formulas, we build a linear regression model (i.e., we use scikit-learn [61]) for each of the generated BFs using the entire dataset. To practically select the best model, i.e., the “best formula”, we consider those ones with the highest R^2^. Once the best formulas have been selected, there is an extra optimization step based on a simple grid search.

Specifically, to further improve the performance of our models, we introduced a “formula optimization” step. In detail, we focus on the top formulas obtained using each one of the two generators, and we use a grid search to find the relative weights of each prototype function of the basic properties (i.e., each f(BP_i_)) within the formula. The grid search ranges between 0.0 and 1.0 with the increasing step of 0.1 used simultaneously for all the weight coefficients (i.e., an exhaustive search through the specified subset of values for the a, b, c, d coefficients is simultaneously performed). We multiply each f(BP_i_) of the formula by the weight coefficient, and we optimize the final R^2^ value. It is important to note here that for each set of weight coefficients generated during the grid search, we build a new linear regression model. Thus, at each step of the grid search, we are updating the weight coefficients, as well as the slope and intercept coming from the linear regression. The final shape of the generated formulas corresponding to potential SFs will be, accordingly to the generators used, the following:

SF_1_ = m × {[***a***f(BP1) * ***b***f(BP2)]/[***c***f(BP_3_)]} + q (14)

SF_2_ = m × {[***a***f(BP_1_) * ***b***f(BP_2_)]/[***c***f(BP_3_) * ***d***f(BP_4_)]} + q (15)

where m and q are the slope and intercept coming from the LR, respectively, and *a*, *b*, *c* and *d* are the weights optimized via the described grid search step.

The FG code is available for free (see Data Availability Statement ).

### 3.4. Computational Details

**Figure 8:**
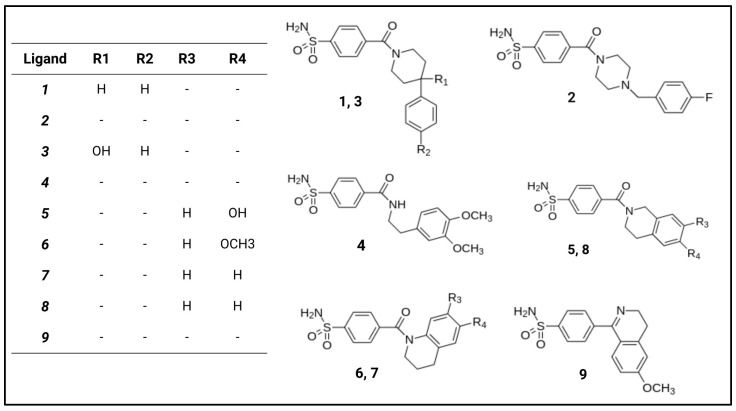
2D structures of hCA II inhibitors investigated in this work.

The geometry of LR complexes between hCA II and benzenesulfonamide ligands were retrieved from the protein data bank with following PDB IDs: 6h2z, 6h34, 6h33, 3v7x, 4z1k, 4z1e, 4z0q, 4z1j and 3vbd [37,38,39]. For the sake of clarity, we refer hereafter to the ligands and to the corresponding LR complexes as **1**, **2**, **3**, **4**, **5**, **6**, **7**, **8** and **9**, respectively. The ligand 2D structures are reported in Figure 8.

The free hCA II structure was obtained from the PDB ID: 1ca2 [62].

The LR complexes and the free hCA II structures were prepared for FMO calculations by using the protein preparation tool [63] followed by geometry optimization using the Powell–Reeves conjugate gradient (PRCG) as implemented in Macromodel [64]. During the optimization, the Zn^2+^ ion, nitrogen atoms of the coordinating His (His94, His96 and His119) and the nitrogen and sulfur atoms of the sulfonamide function were frozen in their X-ray coordinates. For the free hCA II (PDB ID: 1CA2), the crystallization water coordinating the Zn^2+^ ion was maintained during the optimization step, which was then not included in the FMO calculations. This approach was necessary since without any constraint, the sulfonamide group coordinates the Zn^2+^ ion also via oxygen atoms as result of a geometry optimization calculation, drastically altering the binding pose of the ligand with respect to the X-ray structure.

The ligand structure was refined by using the ligand preparation tool [65] and then optimized using the same parameters adopted for LR complexes. For both protein and ligand preparation procedures, the OPLS 2005 FF and the GB/SA effective solvation model (water) were adopted.

Then, for each LR complex, we detected the residues within a range of 6 Å from the ligand, and the final binding pocket was made via the union of residues of all 6 Å binding pockets. The amino acid termini resulting after backbone cutting, -NH and -C=O, were capped by adding H atoms. The reduced LR complexes were built using Python scripts based on ProDy [66,67,68] and Open Babel [69].

Then, the optimized structures of the isolated ligands and of the reduced structures of LR complexes and hCA II were used as input geometry for FMO2 single-point calculations at the RI-MP2/6-311G level of theory.

The protein structures were split in fragments, each one containing a single amino acid. The covalent bond connecting the C_α_ and NH group was selected as the fragmentation point, using the hybrid orbital projection (HOP) treatment for bond detachment [23].

The water solvation effect was simulated by using the PCM [1] method [51], including the repulsion and dispersion terms (relating keywords: idisp = 1, ntsall = 240, method = FIXPVA, icomp = 2, radii = suahf, icomp = 2, icav = 1). To simulate the solvent screening effect, we used the partial screening method [70].

As reported in recent works [27,71], to avoid the overestimation of the embedded charge transfer energy determined based on the presence of a metal atom, we adopted the ESP-PTC approximation using the screened point charges for all atoms (ESP-SPTC). The atomic charges were screened by adopting the gaussian dumping function (a = b = 1) [72].

The Zn atom was treated by adopting the triple-zeta model core potential (MCP-TZP) [73] and considered as a single fragment.

The validity of choice to adopt the reduced structures for FMO calculations was assessed by performing the comparison between the E^INT^ and PIEDA computed at FMO2 calculations considering the entire and the reduced receptor structures of **1**, **2**, **3**, **4** and **9** LR complexes using the 6–31 G basis set and same setup above-mentioned for the remaining part.

Ligands **1**–**9** are all characterized by the anionic benzenesulfonamide group coordinating the Zn^2+^ ion linked to hydrophobic tail bearing aromatic rings, hereafter named F1 and F2, respectively. To assess the role played by these two moieties, we performed additional FMO calculations considering separately the F1 and F2 portions. To do so, each ligand was split into two fragments, and the fragmentation point was the bond connecting the benzoyl C atom with the N/C of the hydrophobic tail. F1 and F2 termini were capped by using -H and -CH_3_, respectively, as shown in Figure S7.

All FMO calculations were performed by using the GAMESS-US package (version: 30 June 2021—R1) [74].

E^INT^, ∆E, FE2 and F2LE values, computed as described above, are different representations of the same quantity, and therefore, only one of them can be included in one SF.

The ligand HIE was computed by adopting the GRID method, and the ligand hydrophobicity was described using the logP (octanol/water) calculated by using Moka [75].

The experimental free binding energies (ΔG_exp_) of the investigated LR complexes (Table S6) were computed starting from K_i_ [37,38,39] according to the following formula:

ΔG_exp_ = −RTln(1/K_i_) = RTlnK_i_ (16)

## 4. Conclusions

The interactions concurring in the stabilization of metalloprotein–ligand complexes are strongly characterized by polarization and CT phenomena that can be evaluated accurately by using QM methods. As a case study, we selected a set of nine small molecules, characterized by a benzenesulfonamide group linked to an aromatic hydrophobic tail, able to inhibit the hCA II enzyme, which contains a Zn^2+^ in the active site. These specific complexes can be profitably studied by means of our FMO/GRID procedure combining the ab initio FMO method, to evaluate the electrostatic and CT interactions, and the GRID approach, to assess the hydrophobic interactions. To reduce the computation burden of FMO calculations, we used reduced models of the LR complexes’ binding core, composed of the ligand and 35 surrounding fragments. We found that the E^INT^ values computed on these reduced LR complexes were highly consistent with the corresponding values computed on the entire receptors (R^2^ = 0.90). These results suggest that the use of reduced LR complexes might be considered a routine approach in FMO-based CADD studies allowing for the assessment of many binding poses at a reduced computational demand.

The FMO energy terms, such as ΔE^FMO2^, E^INT^, F2LE and FE, were combined with hydrophobic interaction energies (HIE and HIE-E) and logP within a machine learning approach, to obtain a final SF formula. While the multilinear regression method was not able to find a satisfying SF to reproduce the experimental binding energies, we evidenced how an FG approach succeeded in finding specific nonlinear relations among HIE-E, logP and F2LE and reproducing the experimental binding free energy with a high accuracy (R^2^ = 0.76, RMSE = 0.34) that was even improved by considering eight over nine ligands (R^2^ = 0.95, RMSE = 0.18).

The mathematical form of the SF obtained by using the FG approach reflected a specific balance between electrostatic and hydrophobic interactions, with the latter playing a key role in determining the binding free energy. These results can be used to support the design of new and potent hCA II inhibitors.

As a final remark, this work shows that the FG approach can be considered a promising machine learning method to develop effective empirical SFs since it permits finding the relative contribution to the final binding free energy of each energy term, eventually non-linearly correlated, supporting the CADD studies, even with a reduced data set.

## Figures and Tables

**Table 1:** ΔE^FMO^, F2LE, E^INT^ and FE values computed for LR complexes formed by ligands **1**–**9** and hCA II. All energy values are in kcal/mol.

ligand	ΔE^FMO^	F2LE	E^INT^	FE
**1**	−37.6	−1.6	−173.2	−7.2
**2**	−53.7	−2.1	−186.2	−7.2
**3**	−37.4	−1.5	−175.5	−7.0
**4**	−42.7	−1.7	−173.6	−6.9
**5**	−61.1	−2.5	−181.1	−7.5
**6**	−36.7	−1.5	−163.2	−6.8
**7**	−67.6	−3.1	−180.1	−8.2
**8**	−70.5	−3.2	−179.3	−8.2
**9**	−38.6	−1.8	−163.8	−7.4

**Table 2:** Computed values for HIE, HIE-E (HIE/number of heavy atoms) and logP.

Ligand	HIE *	HIE-E *	logP
**1**	−38.9	−1.6	0.92
**2**	−37.9	−1.5	−0.01
**3**	−28.1	−1.1	−0.36
**4**	−30.6	−1.2	0.41
**5**	−35.0	−1.5	−0.28
**6**	−24.3	−1.0	0.68
**7**	−32.0	−1.5	0.6
**8**	−30.2	−1.4	0.32
**9**	−34.3	−1.6	0.46

* values in kcal/mol.

## Data Availability

The Formula Generator method has been implemented in open-source software developed in Python by Loriano Storchi, available at https://github.com/lstorchi/formulagenerator (accessed on 17 July 2024).

## Supplementary Materials

The following supporting information can be downloaded at: https://www.mdpi.com/article/10.3390/molecules29153600/s1, Figure S1: scatter plots of FMO energy terms computed considering the reduced and the entire receptor for ligands **1**, **2**, **3**, **4** and **9**; Figure S2: PIE graphs of interactions between ligands **1**–**6** and residues of the reduced hCA II structure; Figure S3: PIE graphs of interactions between ligands **7**–**9** and residues of the reduced hCA II structure; Figure S4: PIE graphs of the interactions of F1 and F2 fragments (ligands **1**–**6**) with the residues in the reduced hCA II structure; Figure S5: PIE graphs of interactions between F1 and F2 fragments of ligands **7**–**9** and residues of the reduced hCA II structure; Figure S6: scoring functions obtained using the MLR approach combining E^INT^ with logP and with HIE; Figure S7: ligand fragmentation scheme adopted for the second run of FMO calculations; Table S1: list of the FMO fragments composed of the reduced LR complex; Table S2: E^INT^ values computed at the RI-MP2/6-31G//PCM [1] level of theory for ligands **1**, **2**, **3**, **4** and **9** using the entire and reduced LR complex; Table S3: EDA of the ligand–Zn^2+^ interaction energy computed using the reduced LR model; Table S4: EDA of the ligand–residue interaction energy computed using the reduced LR complex; Table S5: experimental binding free energy values and all the basic property values used to build the SF; Table S6: binding free energy values derived from experimental K_i_, the number of ligand heavy atoms for each hCA II inhibitor investigated in this work and the PDB IDs of the corresponding LR complexes.
