# Supplementary material for: Combining the Fragment Molecular Orbital and GRID Approaches for the Prediction of Ligand–Metalloenzyme Binding Affinity: The Case Study of hCA II Inhibitors
Source: Molecules. 2024 Jul 30;29(15):3600. doi: 10.3390/molecules29153600 (PMC11313991; doi:10.3390/molecules29153600)
Supplement: Supplementary file 1 [file molecules-29-03600-s001.zip › molecules-3102752-supplementary.pdf]

Supporting Information

Combining the Fragment Molecular Orbital and GRID Approaches for the Prediction of Ligand–Metalloenzyme Binding Affinity: The Case Study of hCA II Inhibitors

Roberto Paciotti *, Nazzareno Re and Loriano Storchi

Department of Pharmacy, Università "G. D'Annunzio" Di Chieti-Pescara, 66100 Chieti, Italy; nre@unich.it (N.R.); loriano@storchi.org (L.S.)

* Correspondence: r.paciotti@unich.it

Contents

Figure S1 2

Figure S2 3

Figure S3 4

Figure S4 5

Figure S5 6

Figure S6 6

Figure S7 7

Table S1 8

Table S2 8

Table S3 9

Table S4 9

Table S5 10

Table S6 10

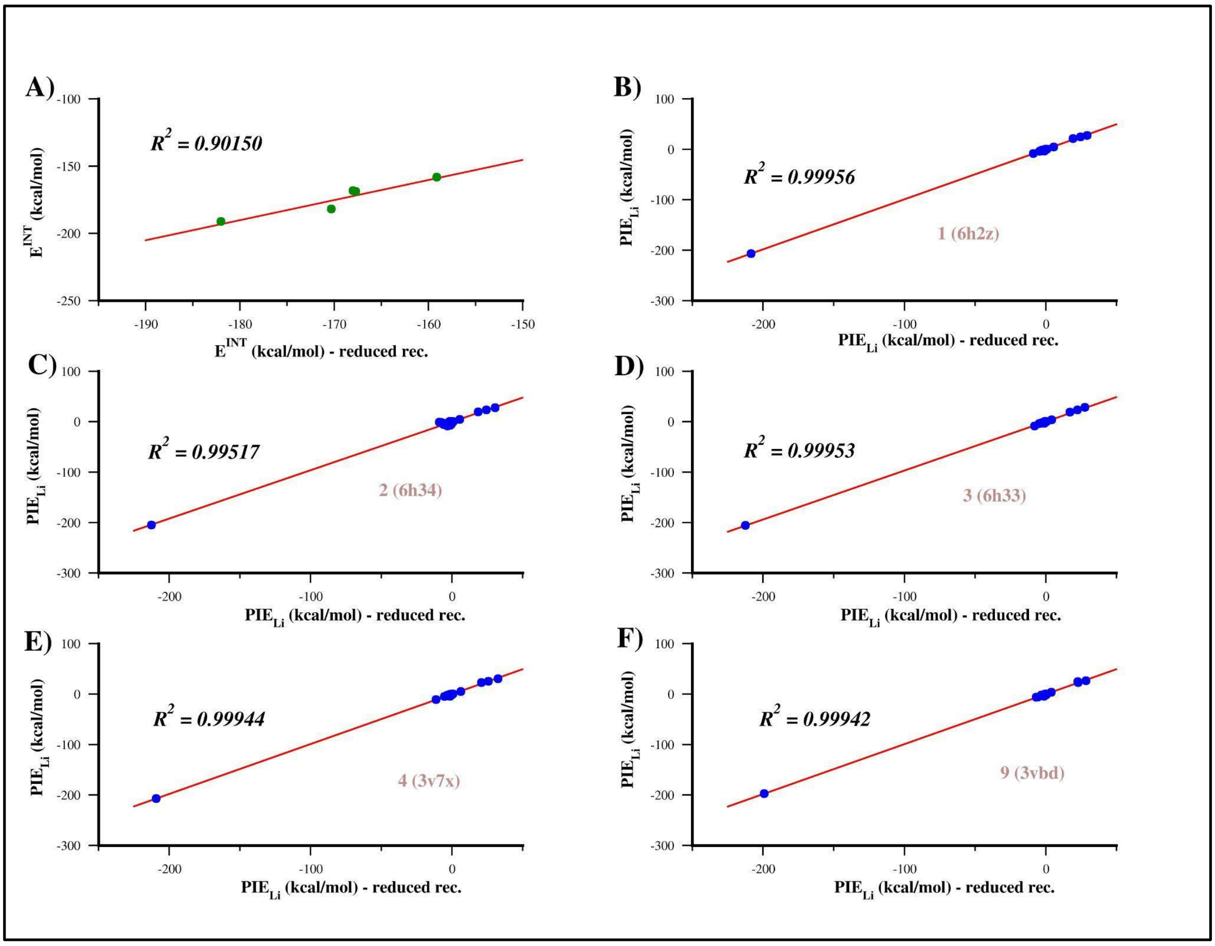

Figure S1. A) Scatter plot of E^{INT} computed considering the reduced and the entire receptor for ligand **1**, **2**, **3**, **4** and **9**. B-F) Correlation between the PIE values computed for the ligand-residues interaction using the reduced receptor (x axis) and the same residues in the entire receptor (y axis) computed for ligand **1** (B), **2**, (C), **3** (D), **4** (E) and **9** (F), respectively. The correlation coefficient (R^2) and line (in red) are also shown.

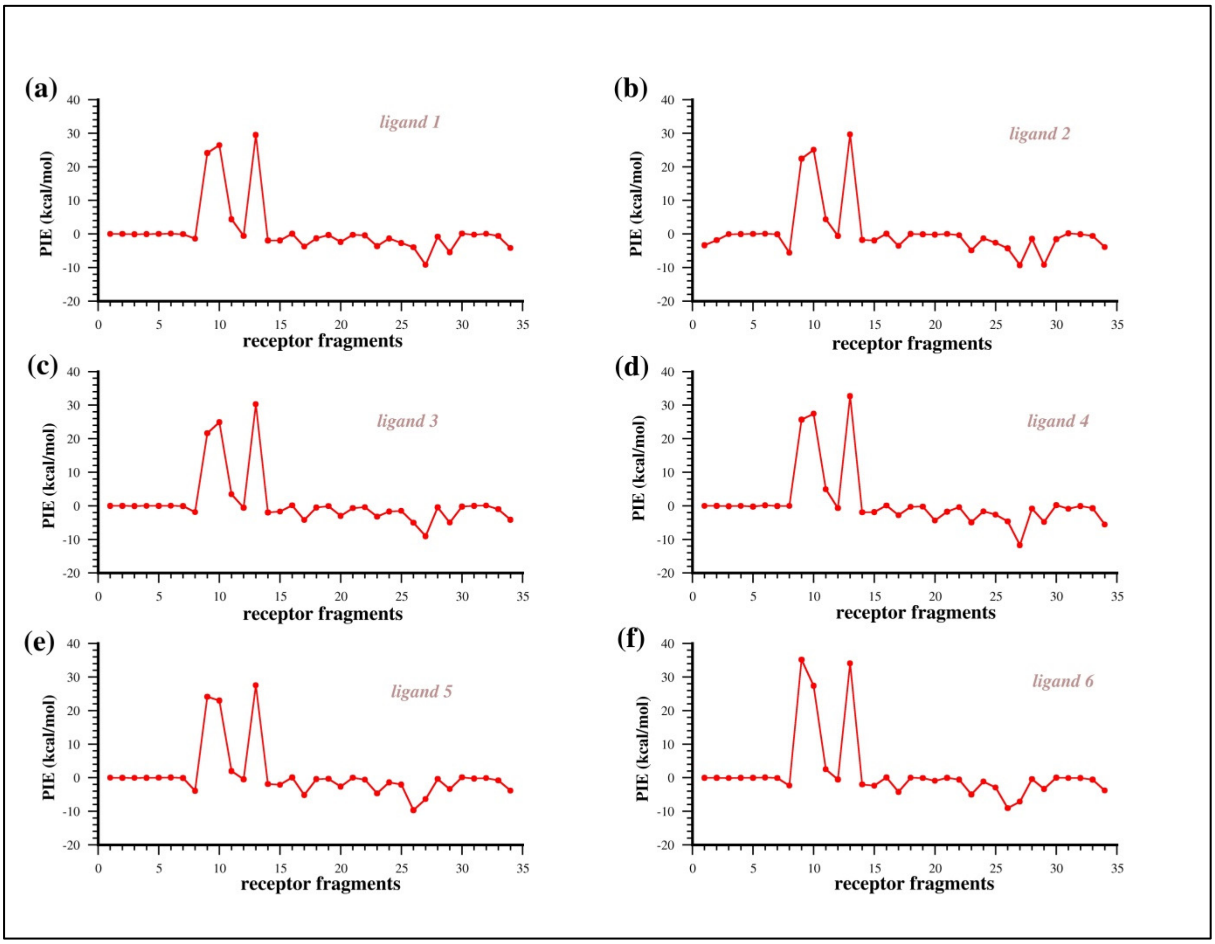

Figure S2. PIE graphs of interactions between ligands **1**–**6** and residues of the reduced hCA II structure (figures a, b, c, d, e and f, respectively).

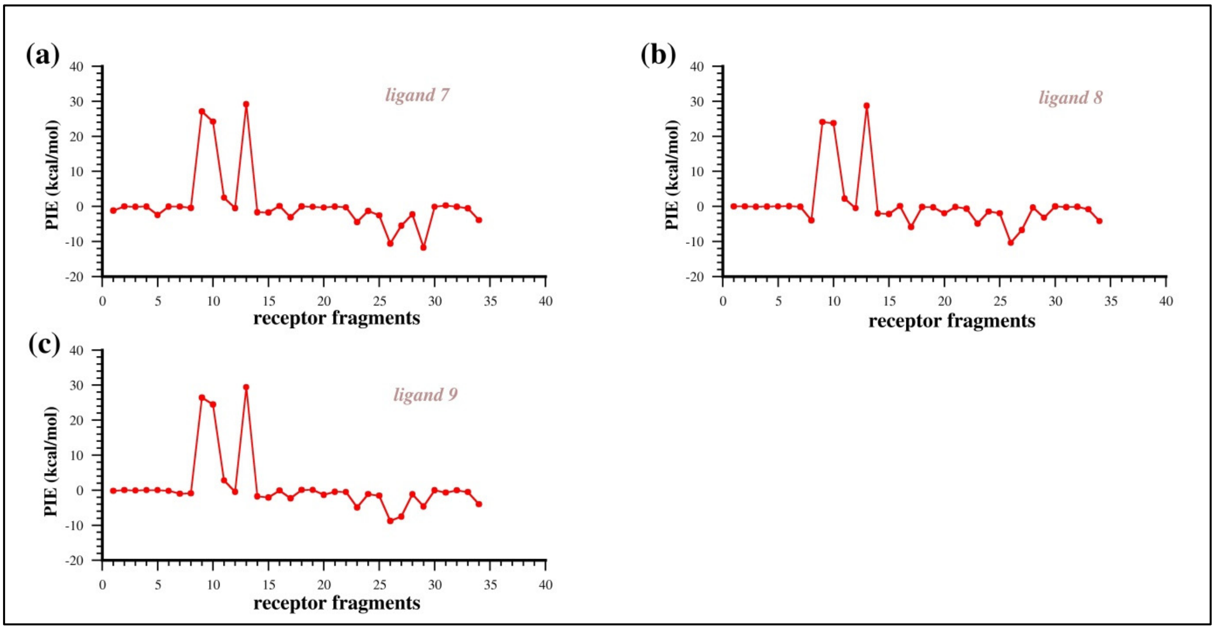

Figure S3. PIE graphs of interactions between ligands **7–9** and residues of the reduced hCA II structure (figures a, b, and c, respectively).

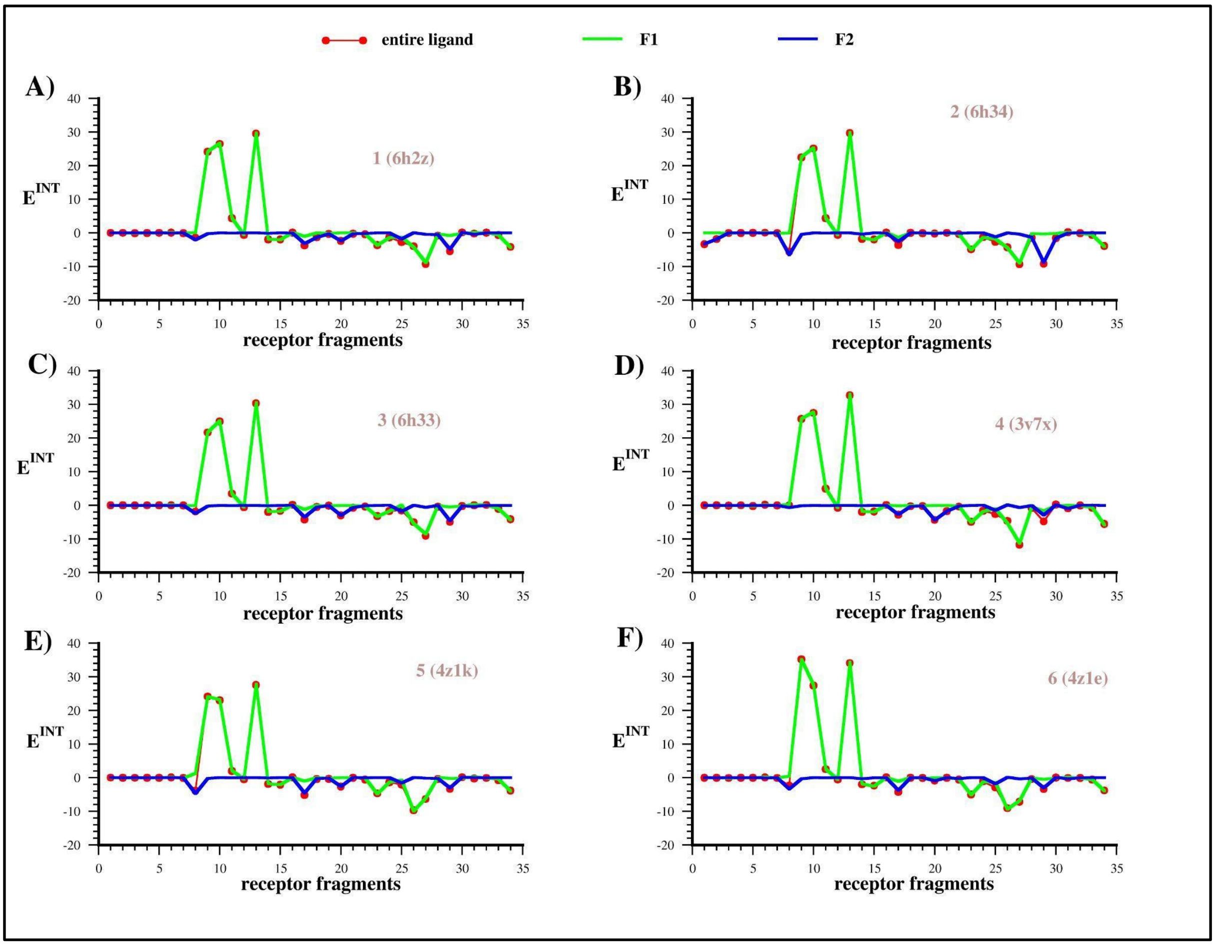

Figure S4. PIE graphs of the interactions of F1 (green) and F2 (blue) fragments (ligands **1–6**) with the residues in the reduced hCA II structure (figures A-F, respectively). The PIE values computed adopting the entire ligand are shown with red dots.

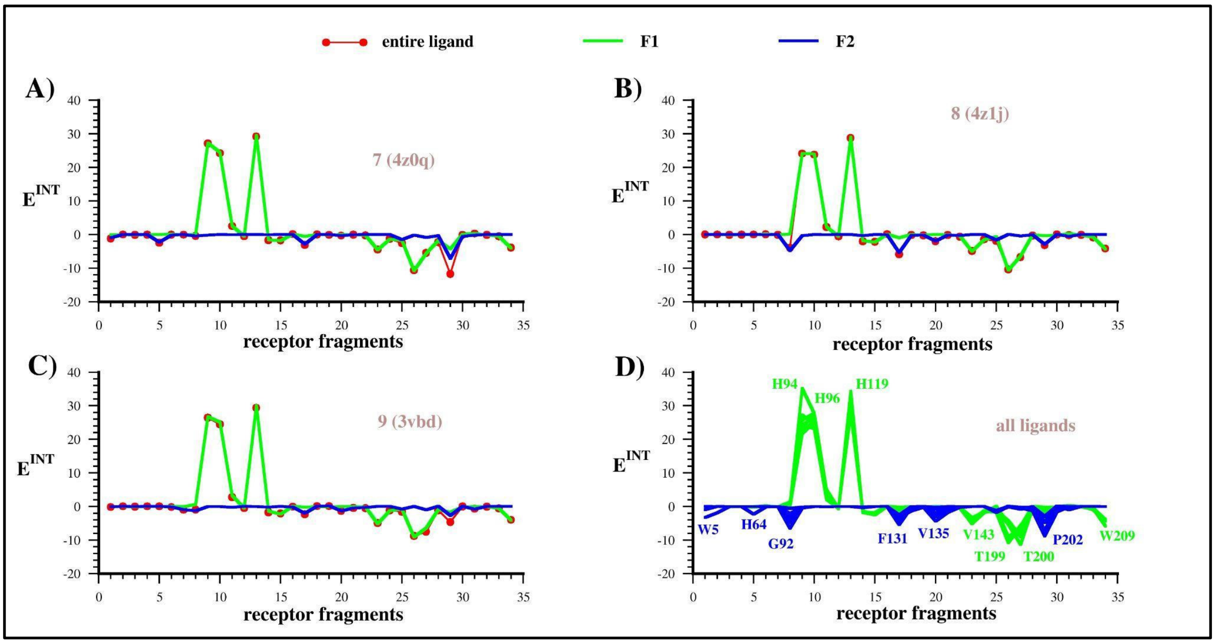

Figure S5. PIE graphs of interactions between F1 (green) and F2 (blue) fragments of ligands **7–9** and residues of the reduced hCA II structure (figures A, B, and C, respectively). The PIE values computed adopting the entire ligand are shown with red dots; D) superposition of PIE graphs computed for F1 (green) and F2 (blue) fragments all ligands, **1-9**, with the most important receptor residues reported by using the one letter code.

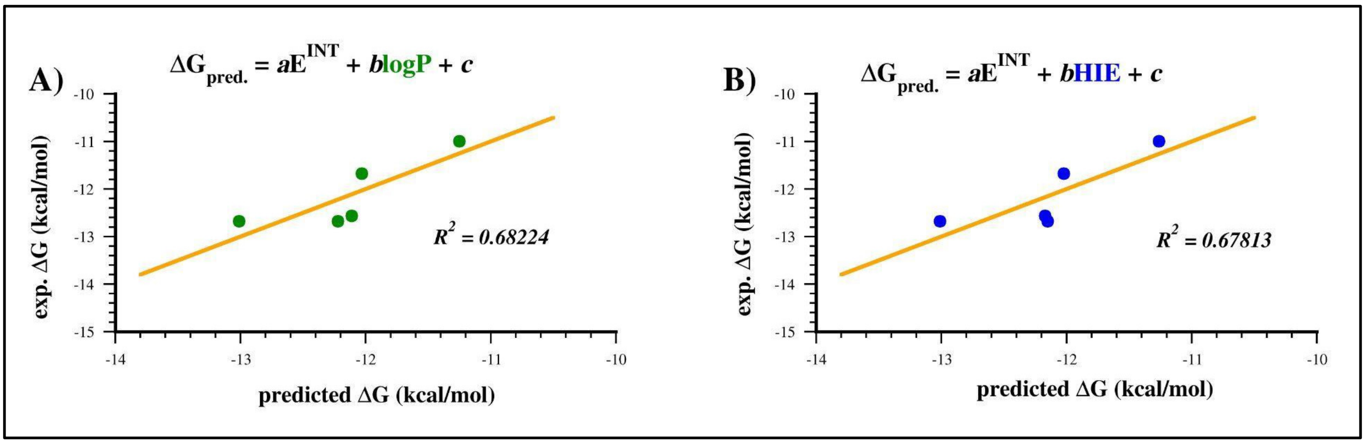

Figure S6. Scoring functions obtained using the MLR approach combining E^{INT} with A) logP (a=0.0808, b = -0.1066, c = 2.0297) and with B) HIE (a = 0.1, b = 0.0003, c = 1.5854) using a reduced data set (complexes **1**, **2**, **3**, **4** and **9**).

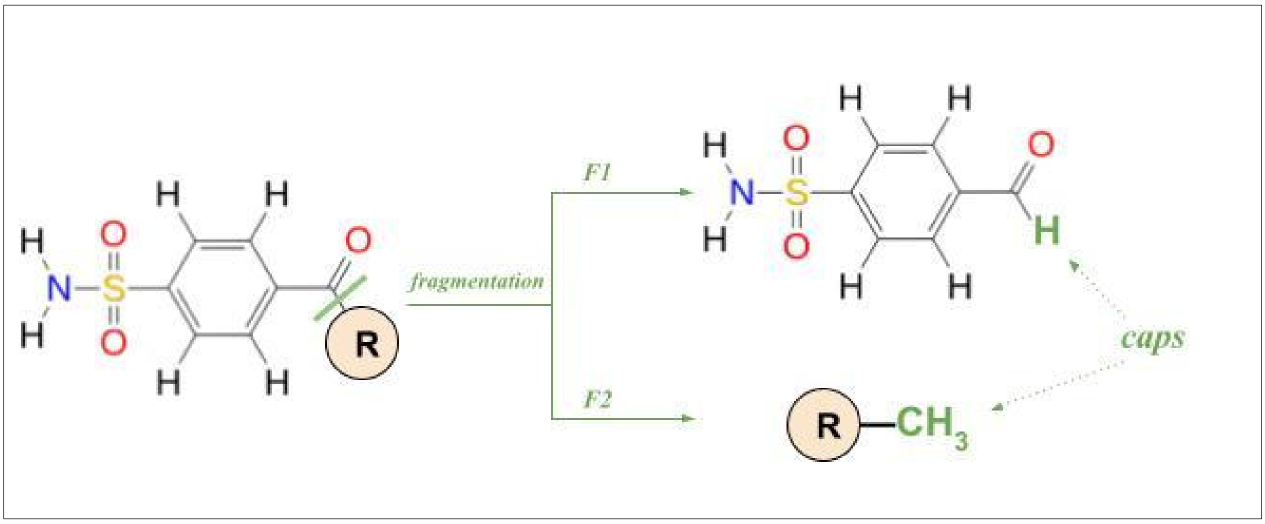

Figure S7. Ligands' fragmentation scheme of adopted for the second run of FMO calculations. The F1 fragment, including the benzene-sulphonamide group, and the hydrophobic tail, F2, are capped by using -H and -CH_3, respectively.

Table S1. List of the FMO fragments composing the reduced LR complex.

Fragment	Description	Fragments	Description
1	Trp5	*19*	Lys133
2	Phe20	*20*	Val135
3	Ser29	*21*	Gln136
4	Asn62	*22*	Leu141
5	His64	*23*	Val143
6	Asn67	*24*	Ser197
7	Ile91	*25*	Leu198
8	Gln92	*26*	Thr199
9	His94	*27*	Thr200
10	His96	*28*	Pro201
11	Glu106	*29*	Pro202
12	His107	*30*	Leu203
13	His119	*31*	Leu204
14	Val121	*32*	Cys206
15	His122	*33*	Val207
16	Asp130	*34*	Trp209
17	Phe131	*35*	Zn^{2+}
18	Gly132	*36*	ligand

Table S2. E^{INT} values computed at RI-MP2/6-31G//PCM[1] level of theory for ligands **1**, **2**, **3**, **4** and **9** using the entire and reduced LR complex.

Ligand	E^{INT} (*entire LR complex*)	E^{INT} (*reduced LR complex*)
1	-168.4	-168.0
2	-191.2	-182.0
3	-182.0	-170.3
4	-168.9	-167.7
9	-158.3	-159.1

Table S3. EDA of the ligand-Zn^{2+} interaction energy computed using the reduced LR model. All energy values are in kcal/mol.

Ligand	E^{INT} §	E^{es}	E^{ex}	E^{ct}	E^{disp}	E^{sol}
1	-210.9	-416.3	106.9	-34.6	-13.1	146.1
2	-208.9	-411.6	109.6	-35.2	-13.2	141.4
3	-209.2	-419.0	115.8	-36.7	-13.3	144.0
4	-211.2	-432.7	139.2	-41.8	-13.7	137.9
5	-207.2	-419.0	117.2	-36.7	-13.4	144.7
6	-215.4	-461.6	155.2	-42.9	-13.4	147.3
7	-208.2	-419.4	109.0	-33.7	-12.9	148.8
8	-205.9	-410.8	107.4	-34.1	-13.1	144.7
9	-200.7	-352.6	139.6	-40.6	-13.2	66.1

§ $E^{INT} = E^{es} + E^{ex} + E^{ct} + E^{disp} + E^{sol}$

Table S4. EDA of the ligand-residues interaction energy computed using the reduced LR complex. All energy values are in kcal/mol.

Ligand	E^{INT} §	E^{es}	E^{ex}	E^{ct}	E^{disp}	E^{sol}
1	37.7	121.8	25.1	-15.5	-43.9	-49.7
2	22.7	100.6	31.6	-19.1	-51.2	-39.1
3	33.7	119.2	24.3	-15.6	-45.1	-49.0
4	37.6	118.8	29.0	-15.3	-47.7	-47.3
5	26.1	103.8	25.3	-16.2	-44.7	-42.1
6	52.2	121.6	26.8	-9.9	-42.1	-44.3
7	28.1	120.0	24.5	-16.3	-43.7	-56.5
8	26.5	104.0	25.9	-15.6	-45.0	-42.8
9	36.9	104.0	25.2	-15.6	-41.9	-34.7

§ $E^{INT} = E^{es} + E^{ex} + E^{ct} + E^{disp} + E^{sol}$

Table S5. Experimental binding free energy values (ΔG_{exp}) and all the basic properties values used to build the SF. All energy terms are in kcal/mol.

Ligand	ΔG_{exp}	ΔE^{FMO}	F2LE	E^{INT}	FE	HIE	HIE-E	logP
1	-12.68	-37.6	-1.6	-173.2	-7.2	-38.9	-1.6	0.92
2	-12.68	-53.7	-2.1	-186.2	-7.2	-37.9	-1.5	-0.01
3	-12.57	-37.4	-1.5	-175.5	-7.0	-28.1	-1.1	-0.36
4	-11.68	-42.7	-1.7	-173.6	-6.9	-30.6	-1.2	0.41
5	-11.36	-61.1	-2.5	-181.1	-7.5	-35.0	-1.5	-0.28
6	-11.24	-36.7	-1.5	-163.2	-6.8	-24.3	-1.0	0.68
7	-11.14	-67.6	-3.1	-180.1	-8.2	-32.0	-1.5	0.6
8	-11.06	-70.5	-3.2	-179.3	-8.2	-30.2	-1.4	0.32
9	-11.00	-38.6	-1.8	-163.8	-7.4	-34.3	-1.6	0.46

Table S6. Binding free energy values derived from experimental K_i, the number of ligand heavy atoms for each hCA II inhibitors investigated in this work, and the PDB IDs of the corresponding LR complexes.

Ligand	ΔG_{exp} (kcal/mol)	# heavy atoms	PDB ID
1	-12.682	24	6h2z
2	-12.682	26	6h34
3	-12.574	25	6h33
4	-11.684	25	3v7x
5	-11.355	24	4z1k
6	-11.241	24	4z1e
7	-11.137	22	4z0q
8	-11.063	22	4z1j
9	-10.997	22	3vbd
